# Influence of radiation on green-synthesized AgNPs and their role in enhancing fluoride stress tolerance in rice

**DOI:** 10.1038/s41598-026-40077-6

**Published:** 2026-03-01

**Authors:** Samreen Kazmi, Nageswara Rao Reddy Neelapu, Ravishankar Kumar Ch, Chagam Koteswara Reddy, Challa Surekha

**Affiliations:** 1 Biochemistry and Bioinformatics Division, Department of Life Sciences, School of Sciences, GITAM (Deemed to be University), Visakhapatnam, A.P. 530045 India; 2 Department of Biotechnology, Mahatma Gandhi University, Narketpally Mandal, Telangana 508003 India; 3 Department of Physics, School of Sciences, GITAM (Deemed to be University), Visakhapatnam, A.P. 530045 India; 4 Microbiology and Food Science Technology Division, Department of Life Sciences, School of Sciences, GITAM (Deemed to be University), Visakhapatnam, A.P. 530045 India

**Keywords:** *Bryophyllum pinnatum*, Fluoride stress, *Oryza sativa*, Silver nanoparticles, Green synthesis, Biochemistry, Plant sciences

## Abstract

Fluoride stress severely impairs the growth and productivity of economically important crops, including rice, resulting in significant yield losses and threatening agricultural sustainability. Enhancing crop resilience requires strategies that balance plant development and stress responses. In this study, we adopted a multi-step approach to mitigate fluoride toxicity in rice. First, soil properties—texture, organic matter, pH, and nutrient content—were analysed to guide crop recommendations and optimize yield. Second, silver nanoparticles (AgNPs) were synthesized using a green approach with *Bryophyllum pinnatum* plant extract and characterized by UV–Vis spectroscopy, FTIR, SEM, XRD and DLS, confirming their structural integrity and morphological changes. Third, the interaction of radiation with *B. pinnatum* plant extract and AgNPs was examined, revealing higher energy absorption in the extract compared to nanoparticles. Finally, plant responses under fluoride stress were evaluated by comparing AgNP priming with conventional foliar fertilizer. AgNP treatment significantly improved germination, root and shoot length, photosynthetic pigments (chlorophyll), and antioxidant enzyme activity, resulting in enhanced growth and yield. These findings demonstrate, for the first time, that AgNP priming effectively regulates fluoride-sensitive defense pathways, offering a promising strategy for developing fluoride-tolerant crops and improving agricultural productivity under stress conditions.

**Supplementary Information:**

The online version contains supplementary material available at 10.1038/s41598-026-40077-6.

## Introduction

Fluoride (F) contamination in groundwater and soil is a significant environmental and health issue in numerous global locations. F contamination often arises from natural sources, such as F-rich minerals, and also results from human activities, including industrial processes and the use of certain fertilizers or pesticides. Manufacturing industries, such as Aluminium smelters, Beryllium extractors, coal-based thermal stations, semiconductor & steel industries, generate fluoride-rich effluents. Fluoride becomes mobile after entering soil, and groundwater; and reaches cultivated crops, fruits, and vegetables, depending on the prevailing geological agents^1^. The negative effect of fluorine on plants is evident in chlorosis and necrosis, which consequently inhibit plant growth and yield. The bioaccumulation of fluoride further affects the food chain via the drinking water route, thereby increasing the risk to human health. The health risks include dental, skeletal, reproductive, developmental, renal, neurological, endocrine, and carcinogenic effects^2–4^.

Soil contamination with F in the Telangana districts of India poses a significant challenge to crop development^5^. Under F stress, the oxidative burst triggers several molecular and biochemical changes that may hinder plant development, ultimately affecting productivity. Therefore, sustainable agriculture in F-stress soils is of utmost importance as F stress impacts the growth and productivity of many economically relevant crops, such as rice (*Oryza sativa*).

Recent research has shown that applying exogenous Salicylic Acid (SA) or nanoparticles (NPs) enhances resistance to F stress^6–8^. The role of NPs in mitigating abiotic and biotic stress is well-studied and reported^9,10^. The use of biogenic silver nanoparticles (AgNPs) to minimize biotic stress in rice plants against *Aspergillus flavus* and in tomato plants against *Botrytis cinerea* was reported by Sultana et al.^9^and Anum et al.^11^. The silver, silicon, and zinc oxide nanoparticles are used to mitigate abiotic stresses like drought, fluoride, heat, and salinity^7–10^. Waqas nano Mazhar et al.^10^used zinc oxide nanoparticles to prime the seeds of rice to mitigate drought stress. Similarly, SiNPs and AgNPs were used to mitigate abiotic stresses, such as fluoride in rice^8^and pigeonpea^7^, as well as heat and salinity in wheat^12,13^. Yadu et al.^7^reported that the exogenous application of AgNPs provided tolerance against F-toxicity by enhancing the levels of proline, reactive oxygen species (ROS), total and reduced glutathione, and glyoxalase I and II activities. This plethora of reports shows that NPs can mitigate abiotic and biotic stress, especially F stress.

Furthermore, the use of NPs as biofertilizers in agriculture is increasing. The AgNPs and Zinc oxide (ZnO) NPs are used as biofertilizers to improve the growth and yield of maize and peas, respectively. Tondey et al.^14^coated fodder maize seeds with ZnO NPs synthesized by wet-chemical methods to improve vegetative growth, yield, and quality. Rahman et al.^15^treated pea seeds with AgNPs synthesized by the sodium borohydride (NaBH_4_) reduction technique and reported enhanced growth and yield. Due to their high surface area, high fraction of surface atoms, and other effects, AgNPs have emerged as a potential candidate for mitigating stress and enhancing crop yield. Therefore, the present objective is to analyze soil samples for their physical, and chemical characteristics, to synthesize and characterize green AgNPs using plant aqueous extracts of *Bryophyllum pinnatum*, to study the impact of energy absorption/radiation on plant extract and AgNPs, and to investigate the effect of AgNPs on rice plants in combating F stress.

## Materials and methods

Study area and soil sampling - The Narkatepalli basin lies 20 km from Nalgonda town and covers 65 sq km. The Narkatepalli basin originates in catchment areas, and its streams flow into the southern region. It flows from NWn to SEn to join the Bhimasamudram Cheruvu at Cherlapalli in Nalgonda district, Telangana, India. The area consists of 19 villages and 15 natural ponds. Surface soil samples (0–15 cm) were collected randomly from 10 regions in the Narketpalli area, Nalgonda Dist, Telangana. The samples were brought to the lab in polyethylene bags with ice packs. Samples were processed or stored at 4 °C for 18 to 24 h. The samples were labeled accordingly as S1, S2, S3, S4, S5, S6, S7, S8, S9 and S10 (where S1- Goplaipalli, S2- Laxmi puram, S3-Yenuguladhari, S4-Chaudampalli, S5- Kodapakagudem, S6-Serbaigudem, S7- Yellareddy gudem, S8-Ramnagar colony, S9-Shankarnagar, S10-Chinnanarayanapur). These samples were air-dried, ground, and separated via a 2 mm sieve. The samples were further assessed for pH, organic content, and physico-chemical characteristics.

Physiochemical analysis of soil samples - The soil samples were characterized using a standard procedure of APHA^16^. The soil sample (40 g) was air-dried, then added to 100 mL of distilled water and mixed thoroughly. The soil was then analyzed for its physical characteristics, such as pH and electrical conductivity, using an electrode-equipped pH meter and an electrical conductivity meter, respectively.

The chemical analysis of the sample was performed according to the procedure described by Subbiah and Asija^17^for nitrogen (N). The Jackson^18^method was used to estimate phosphorus (P), potassium (K), and magnesium (Mg), whereas the Yoshida et al.^19^method was employed to assess calcium (Ca) in the sample. The Piper^20^method was used to determine Zn, Cu, iron (Fe), and manganese (Mn). The APHA^16^standard procedure estimated F using an ion-selective meter (Mettler Toledo MA 235 pH/ion analyzer). The total ionic strength adjustment buffer (TISAB) was utilized to maintain an appropriate strength and prevent complex formation.

The analysed soil exhibited a moderately alkaline pH (7.86), which can restrict the solubility of essential micronutrients such as iron, manganese, and zinc. Additionally, the moderate level of organic carbon supports basic microbial and nutrient cycling functions, but may not be sufficient to fully buffer against abiotic stress, such as fluoride toxicity. These physiochemical properties indicate a soil matrix that challenges nutrient bioavailability and plant growth. Hence, the site provides a relevant platform for evaluating the role of biofabricated AgNPs in enhancing nutrient uptake and improving plant resilience under fluoride-stressed conditions.

### Green synthesis and characterization of AgNPs

*B. pinnatum* was collected from the GITAM greenhouse, Visakhapatnam, and was authenticated by taxonomist Dr. K. Vijaya Lakshmi (Retired Professor), Department of Biotechnology, GITAM School of Technology, GITAM (Deemed to be University), Visakhapatnam. AgNPs were synthesized by taking 35 g of sterile, chopped leaves of *B. pinnatum* and cooking them in 220 ml of distilled water until the liquid turned dark yellow. The solution was filtered through Whatman No. 1 filter paper, and the resulting plant extract was chilled to 4 °C for subsequent use. A mixture of 4 ml of *B. pinnatum* plant extract and 36 ml of 1 mM silver nitrate (AgNO_3_) was incubated under sunlight for 12 h and in the dark for 60 h, without stirring. This incubation led to the formation of polydisperse AgNPs. The AgNPs were separated by centrifuging at 49.054 g for 30 min, resuspended in ultrapure water, and characterized for morphological and biophysical features as per standard protocol^21,22^. Measurements of synthesized nanoparticles were analyzed with experimental methods (i) UV-Visible spectroscopy- Shimadzu Model: UV-2600i for homogeneity, (ii) Fourier Transform infrared spectroscopy (FTIR)- Agilent portable 4300 with shifts in functional and finger print regions, (iii) Dynamic light scattering (DLS) with Nanoparticle Analyzer Horiba-SZ-100 for analysing particle size, (iv) Confirmation of structures by powdered-X-ray diffraction with Brucker D8 Advance, (iv) FESEM-Tescan MIRA S6123 for morphology and fraction of elements.

### Effect of radiation

The effects of radiation were assessed at low doses (Beta radiation) and high doses (gamma radiation) on the *B. pinnatum* plant extract and its synthesized Silver nanoparticles (AgNPs). Studies were performed with Photon Shielding and Dosimetry (PSD) (Phy-X/PSD) to calculate parameters linear attenuation coefficients (LAC), mass attenuation coefficients (MAC), effective atomic number (Zeff), electron density (Neff), energy absorption buildup factor (EABF), and exposure buildup factors (EBF) with software at https://phy-x.net/PSD.

#### Seed treatments

Seeds of rice variety TN1 (genetically stable, susceptible to abiotic stresses and short lived) were obtained from the Directorate of Rice Research (DRR), Indian Council of Agricultural Research, Rajendranagar, Telangana, Hyderabad, India. Seeds were washed thoroughly with detergent and surface-sterilized with 0.1% (w/v) mercuric chloride (HgCl_2_) for 10 min. Then, seeds are rinsed 4–5 times in sterile distilled water and used as an experimental source. Seeds were treated with AgNPs (50 mg/l) and fertilizer (NPK), sown separately in 7 × 7 experimental trays with normal and fluoride soils, and used in all experiments^9^. Seeds that are not treated are kept separately as a control in 7 × 7 trays. All the experiments were carried out in replicates. A sample size of ten was used to determine the plant growth, biochemical and agronomical parameters.

#### Determination of plant growth parameters

Plant growth parameters, such as germination percentage (G %), root length (RL), shoot length (SL), and Seed vigour index (SVI), were determined at regular intervals to assess the efficacy of treated seeds. The RL and SL were calculated using a scale, while G% and SVI were calculated using the following formulas^23^.

G % = [Number of seeds germinated/Total number of seeds] × 100 SVI = G% × [SL + RL]

#### Determination of biochemical parameters

The plant biochemical parameters, including chlorophyll, antioxidant enzymes (superoxide dismutase, catalase, peroxidase), and total phenols, were estimated.

#### Chlorophyll content

The spectrophotometric method was used to determine chlorophyll (Chl) content in the leaves. 0.2 g of the leaves from each sample were ground in 10 ml of 80% acetone. The filtrate was collected into test tubes, and the spectrophotometer was used to measure absorbance at 645, 652, and 663 nm^24^. The following formulas are used to estimate Chl a, Chl b, and total Chl.

$$\hbox{}{\text{Chl a}}=\left\{ {\left( {{\mathrm{12}}.{\mathrm{7}} \times {\mathrm{O}}.{\text{ D 663}}} \right) - \left( {{\mathrm{2}}.{\mathrm{69}} \times {\mathrm{O}}.{\text{ D 645}}} \right)} \right\} \times {\mathrm{V}}/{\mathrm{1}}000 \times {\mathrm{W}}$$

$${\text{Chl b}}=\left\{ {\left( {{\mathrm{22}}.{\mathrm{9}} \times {\mathrm{O}}.{\text{D 645}}} \right) - \left( {{\mathrm{4}}.{\mathrm{68}} \times {\mathrm{O}}.{\text{D 663}}} \right)} \right\} \times {\mathrm{V}}/{\mathrm{1}}000 \times {\mathrm{W}}$$

$${\text{Total chl}}=\left\{ {\left( {{\mathrm{2}}0.{\mathrm{2}} \times {\mathrm{O}}.{\text{D 645}}} \right)+\left( {{\mathrm{8}}.0{\mathrm{2}} \times {\mathrm{O}}.{\text{D 663}}} \right)} \right\} \times {\mathrm{V}}/{\mathrm{1}}000 \times {\mathrm{W}}\hbox{}$$

#### Assay of antioxidant enzyme activity, proline and malonaldehyde (MDA) content

The plant material (1 g) was ground in 10 ml ice-cold 50 mM potassium phosphate buffer (pH 7.8) in a pre-chilled pestle and mortar. The plant extract was centrifuged at 10,000 rpm for 10 min at 4 °C, and the supernatant was used as an enzyme source to estimate antioxidant enzymes, including superoxide dismutase (SOD), catalase (CAT), and peroxidase (PO). SOD assay was performed according to the procedure of Ullah et al.^25^. The percent inhibition of NBT reduction is calculated using the formula “control OD-Sample OD/control OD x 100”, and 50% inhibition of NBT is expressed as 1 unit of the enzyme. The CAT assay was performed according to the procedure described by Ullah et al.^25^. The CAT activity was expressed in nmol of H_2_O_2_ utilized mg-1 protein min-1. One unit is equivalent to a 0.01 decrease in absorbance at 240 nm/mg protein/min. The PO activity was determined as described by Lagrimini et al.^26^. Enzyme activity was expressed as changes in absorbance min-1 mg-1 protein. The total phenol content of extracts was determined using the Folin-Ciocalteau (FC) method^27^. Proline and malonaldehyde content was estimated from plants following the standard protocols^28,29^.

#### Determination of agronomical parameters

The current study analysed the yield profile of rice plants by counting the number of tillers, panicles, spikelets, and rice yield (g) per pot on each plant. A manual electronic balance was employed to determine the weight profile^10^.

#### Statistical analysis

A sample size of ten (*n* = 10) was used to determine the plant growth, biochemical and agronomical parameters. Seeds treated with AgNPs and fertilizer were sown separately in 7 × 7 experimental trays. Untreated seeds are kept separately as a control in 7 × 7 trays (*N* = 49). All the experiments were carried out in replicates. A sample size of ten (*n* = 10), including R1 = 3, R2 = 3, and R3 = 4, and the same size of control group were used to determine all the parameters. The results are represented in mean and standard deviation for growth parameters (G %, RL, SL, SVI), biochemical parameters (Chl content - Chl a, Chl b, and total Chl; enzymes - SOD, CAT, and PO; total phenols), and agronomical parameters (tillers, panicles, spikelets, and rice yield). The statistical significance of the growth, biochemical, and agronomical parameters was assessed between the plants treated with AgNPs and control, grown in normal and F soil using the analysis of variance (ANOVA). The values were considered significant when *P* < 0.05.

## Results and discussion

The study primarily focuses on AgNPs (50 mg/l) that promote biochemical and physiological changes in rice plants and improve agronomic traits, thereby benefiting the control of F stress in the Nalgonda region of Telangana. The current study was conducted in four stages; in the first stage, the soil samples from the Nalgonda region were analyzed for their physical, and chemical, composition. In the second stage, the green synthesis and characterization of AgNPs were investigated. In the third stage, the impact of energy absorption / radiation on plant extract and AgNPs were investigated. In the fourth stage, the effects of AgNPs on rice plants grown in both normal and F-stressed soils were investigated.

### Physical and chemical evaluation of soils in the Nalgonda region

The Nalgonda region has a widespread, pitiful incidence of fluorosis in irrigated soils due to high levels of fluorides in the groundwater. As a result of F contamination in the agricultural system, crop growth is hampered, resulting in lower yields. Table 1 lists the basic physical and chemical characteristics of soil. The results demonstrate that the examined chemical and physical attributes exhibit extensive variation. The value of F in the given soil samples of the Nalgonda region ranges from 4 ppm to 10 ppm and when compared to normal levels (0.3–3 ppm), it’s high. F significantly hinders photosynthesis and other processes. Over time, accumulation might occur gradually and it will travel from roots through stomata or the transpiration stream and gather at the margins of leaves. Thus, F poisoning builds up in the foliage of plants. Plants sensitive to F usually show signs of leaf burn or damage^28^^–33^. F concentrations in plants are generally higher when F concentrations in the environment are higher. At low levels of contamination, F is a common, non-biodegradable, and relatively persistent pollutant that can cause serious health issues that are difficult to treat. Increased soil F levels will increase its accumulation in plants and in food, ultimately affecting human health. The F level in the soil samples from the Nalgonda region is high and higher F levels in paddy plants hinder photosynthesis, inhibiting growth.

**Table 1:** Physical and chemical properties of soil samples in and around Nalgonda region.

S.no	Name of the parameters	S_1_	S_2_	S_3_	S_4_	S_5_	S_6_	S_7_	S_8_	S_9_	S_10_
1	pH	7.69	7.56	7.26	7.58	7.52	7.98	8.26	7.45	7.68	7.62
2	Electrical conductivity (dsm^− 1^)	0.41	0.26	0.16	0.23	0.23	0.95	1.26	0.52	0.46	0.46
3	Organic matter (%)	0.39	0.42	0.44	0.56	0.22	0.46	0.58	0.54	0.45	0.39
4	Available nitrogen (mg/kg)	98.5	95.6	89.6	93.5	96.3	110.2	126.3	142.6	109.2	122.3
5	Available phosphorous(mg/kg)	4.25	3.50	2.75	2.69	3.25	4.56	4.69	4.58	5.25	4.50
6	Available potassium (mg/kg)	140	135	125	115	111	145	163	145	180	179
7	Available zinc (ppm)	1.15	0.85	0.56	0.56	0.63	0.96	1.23	1.25	1.19	1.12
8	Available copper (ppm)	1.10	0.98	0.56	0.45	0.85	1.26	1.23	1.36	1.12	1.26
9	Available iron (ppm)	7.52	4.85	3.65	2.69	2.65	4.56	4.56	4.69	9.58	9.63
10	Available manganese (ppm)	3.68	3.26	1.69	1.59	1.69	2.69	3.15	3.68	3.25	3.56
11	Cation exchange capacity (C. mole proton^+^/kg)	23.5	22.5	15.6	15.6	12.3	19.6	18.6	20.3	24.6	23.2
12	Calcium (mg/kg)	11.2	10.8	6.9	6.9	6.5	9.6	9.6	10.6	14.6	14.2
13	Magnesium (mg/kg)	9.6	9.5	5.9	6.2	6.5	7.6	8.6	8.6	12.6	11.2
14	Sodium (mg/kg)	2.18	2.16	0.96	0.56	0.56	1.56	1.69	1.58	2.75	2.22
15	Potassium (mg/kg)	0.22	0.18	0.21	0.23	0.21	0.26	0.36	0.36	0.26	0.18
16	Fluoride (ppm)	6	4	7	8	5	7	10	10	7	5
17	TNC	**70**	**71**	**75**	**40**	**77**	**59**	**80**	**72**	**64**	**67**

Along with F, other physical and chemical parameters are investigated to understand their effects on plant growth. The soil samples’ physical parameters, like pH, electrical conductivity, and organic matter, are evaluated. The soil pH is slightly alkaline (7.45 to 8.26), which is below the optimal pH range (6.0-7.5) for the plants, and plants growing in soil with excessively high or low pH levels may experience toxicities or nutrient shortages. The electrical conductivity of the soils is low (0.16 to 1.95 dsm-1), indicating a low nutrient concentration. The percentage of organic matter ranged from 0.22 to 0.58% across the different cultivated soil horizons chosen for the study, suggesting that more water can be retained after rainfall. This variation in organic matter serves as an indirect indicator of baseline soil health, which influences crop responses to abiotic stress and external treatments such as AgNPs. The soil’s pH and electrical conductivity indicate that plant growth may be hindered, whereas the soil’s organic matter suggests that plant health is promoted. Although the soil parameter of organic matter supports plant growth, pH and soil electrical conductivity hinder it.

The N: P:K ratio is the primary factor determining soil productivity. The N available in the soil samples of the Nalgonda region ranged from 89.6 to 142.60 mg/kg, which is less than the normal N levels (200–5000 mg/kg) in the soil. Low soil N levels lead to stunted plant growth. Available P in cultivated soil samples from the study area varied from 2.69 to 5.25 mg/kg. P levels in the soil are below the normal range (100–2000 mg/kg), and inadequate P delays plant maturity and reduces yields. The research area’s cultivated soil samples had available K ranging from 111 to 179 mg/kg. K levels in the soil samples are below the normal range (1700–33000 mg/kg), and K scarcity reduces plant growth. In short, a deficiency of NPK leads to reduced plant growth.

Evidence of other trace elements, such as Zn, Cu, Fe, and Mn, ranges from 0.45 to 9.63 ppm. The range of Zn availability among all the trace elements is 0.56 to 1.25 ppm. The Zn level is below the normal range (10–250 ppm), and Zn deficiency leads to yellowing leaves, stunted growth, and poor flowering or fruiting, resulting in low yield. On the other hand, the Nalgonda soils have the lowest Cu availability, ranging from 0.45 to 1.36 ppm. Plants require Cu (5-150 ppm), and the Nalgonda soils have the lowest Cu availability (0.45–1.36 ppm). Cu deficiency reduces plant growth and can make plants more vulnerable to pathogen-causing diseases, such as ergot, which reduces the yield of small grains. Mn availability ranges from 1.59 to 3.68 ppm. Mn is a micronutrient necessary for plant growth and development. Photosystem II (PSII) catalyzes the water-splitting reaction, and the metal is an essential cofactor for the oxygen-evolving complex (OEC) of the photosynthetic machinery. The Mn availability ranges from 1.59 to 3.68 mg/kg, below the normal soil range (200-10000 mg/kg). Mn deficiency reduces crop yields due to impairment of photosynthesis and starch synthesis impairment. Fe availability is high for all trace elements, ranging from 2.65 to 9.63 ppm. Deviation from the normal soil range (5,000–50,000 ppm) indicates iron deficiency. Fe deficiencies significantly impact plant growth and development, and excessive Fe within cells is also harmful. The Fe content in the soil samples is low; therefore, Fe deficiency significantly impacts plant growth and development. While sodium (Na) is not necessary for plants, it is utilized in small amounts to support metabolism and chlorophyll synthesis, much like micronutrients. In the soil samples from the Nalgonda region, the Na concentration ranges from 0.56 mg/kg to 2.75 mg/kg. High sodium reluths in wilted foliage and stunted plant growth and can be due to plants with excessive salt in the soil cannot absorb water, leading to their tissues becoming dry and discoloured. Plants may grow slowly, but not noticeably, when salinity is high.

The total negative charge in soil is measured by the Cation Exchange Capacity (CEC), which can adsorb cations such as Ca, Mg, and K, which are essential for plant nutrition. Therefore, the CEC of soil describes its capacity to supply nutritional cations to the soil solution, which plants can then uptake. The CEC value of Nalgonda region soils ranges from 12.3 to 23.5 cmol/kg. The major essential elements required for plant growth and development include potassium (K), calcium (Ca), and magnesium (Mg). K aids in the formation and movement of carbohydrates, sugars, and oils in plants, boosts plant vigor and disease resistance, and can enhance total yield. The available K value ranges from 0.18 to 0.36 mg/kg, which is lower than the normal soil K range (1700–33000 mg/kg). The tendency of K-deficient plants to wilt on dry, sunny days makes them easily identifiable. The plant appears to be drooping or wilted overall. Deficient plants will appear stocky and have short internodes. Smaller leaf blades and restricted growth are characteristics of younger leaves. The development of leaves, the health of existing roots, and the generation of new roots and root hairs depend on calcium. In the given soil samples of the Nalgonda region, the value of Ca ranges from 6.5 mg/kg to 14.6 mg/kg and is less than the normal range of Ca (700-36000 mg/kg) in the soil. Younger leaves and tissues exhibit the first signs of Ca deficiency, leading to growth inhibition and a bushy appearance. Typically, brown chlorotic spots appear on the margins of the youngest leaves, which are small and malformed, and eventually come together in the centre of the leaf. Mg is essential for photosynthesis and is a major component of chlorophyll, which gives plants their green color. In the given soil samples from the Nalgonda region, the Mg values range from 5.9 mg/kg to 12.6 mg/kg, which is lower than the normal soil Mg range (1200–15000 mg/kg). Plant growth is poor and stunted due to a lack of Chl, leading to Mg deficiency. Thus, the knowledge gained from this study provides robust evidence of the health of the F-rich soil and its effect on plant growth. The study concludes that alterations in soil physical and chemical parameters do not support plant growth. Therefore, developing strategies and tactics to optimize these resources is necessary. Two strategies can be used to alter the soil’s fluoride content: adding amendments that improve soil quality for plant growth, or treating plant seeds to reprogram their ability to withstand or mitigate stress.

### Green synthesis and characterization of AgNPs

**Fig. 1:**
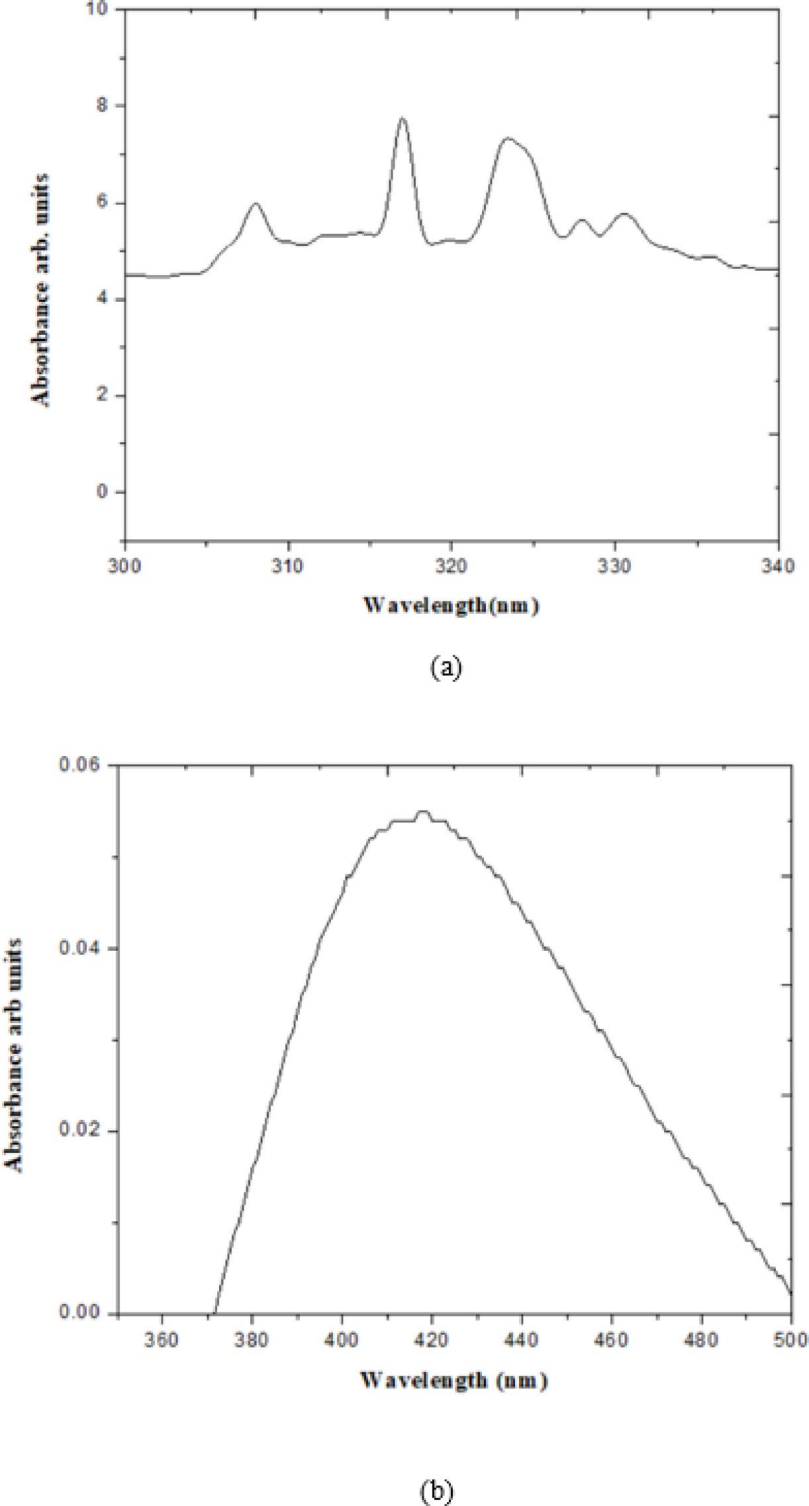
(**a**): UV spectra of leaf extract of *B. pinnatum*. (**b**) UV spectra of synthesised AgNPs from leaf extract of *B. pinnatum*.

The green synthesis of AgNPs was successful using an extract of *B. pinnatum* leaves and an aqueous AgNO_3_ solution. With the addition of leaf extract, the colour of the reaction mixture

changed from light yellow to a yellowish-brown, indicating the generation of AgNPs, which can be explained based on the activation of surface plasmon resonance (SPR) bands^25^. UV spectra of plant extract alone exhibited shoulder peaks distributed around 8 nm centered at 317 nm as observed in Fig. 1(a) indicating nearly spaced electronic transitions within the molecule. Analysing UV spectra of synthesized AgNP attributed with homogenous peak centered at 412 nm (Fig. 1b). Generally, AgNPs show a prominent and broad peak between 380 and 450 nm region using UV-visible spectroscopy. Studies of UV spectra with isolated peak support the literature and the result was similar to Rahman et al.^15^, and which can be explained by the percent reduction of Ag + ion to AgO, determined by SPR intensity. The optical absorption or extinction coefficient of 0.054 at 412 nm, corresponding to the SPR peak, is considered as 100% reduction. The SPR peak of the NPs has been thoroughly established and acknowledged, with a size range of 2–100 nm^32,33^. Synthesis of spherical nanoparticles is due to phytochemicals^33^of plants (may be phenolic/polyphenolic/flavonoids), which are efficient in reducing metal ions to the nanoscale with a unique peak in UV spectra corresponding to maximum absorption^34–36^.

**Fig. 2:**
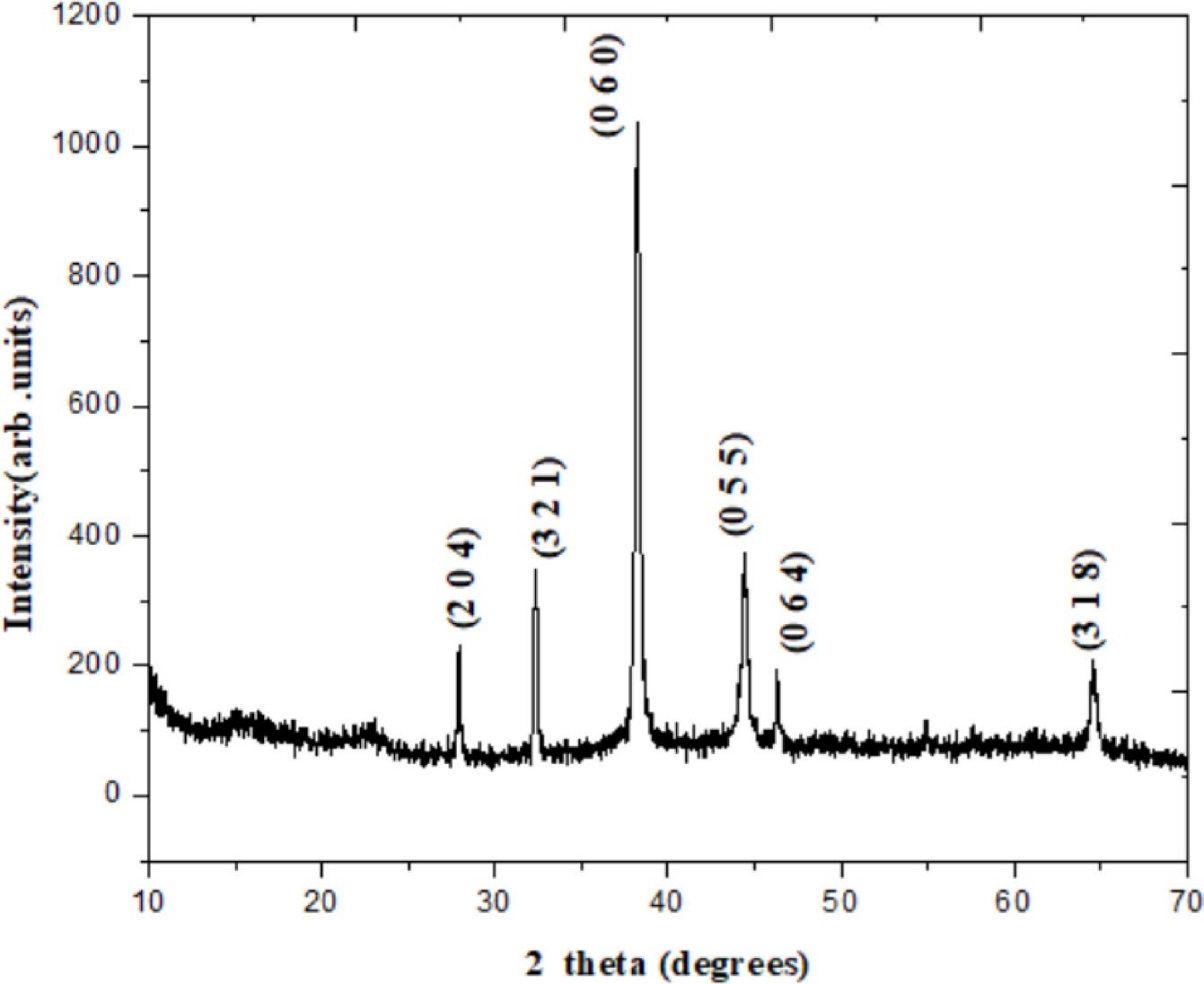
X-ray diffraction spectra of synthesised AgNPs from leaf extract of *B. pinnatum*.

**Fig. 3:**
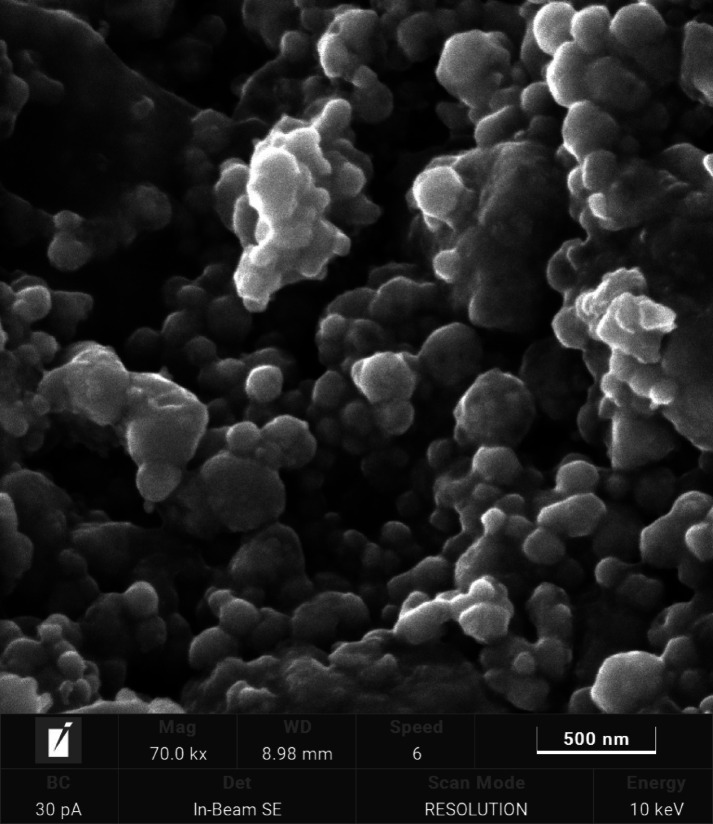
Morphology of synthesised AgNPs from leaf extract of *B. pinnatum*.

**Fig. 4:**
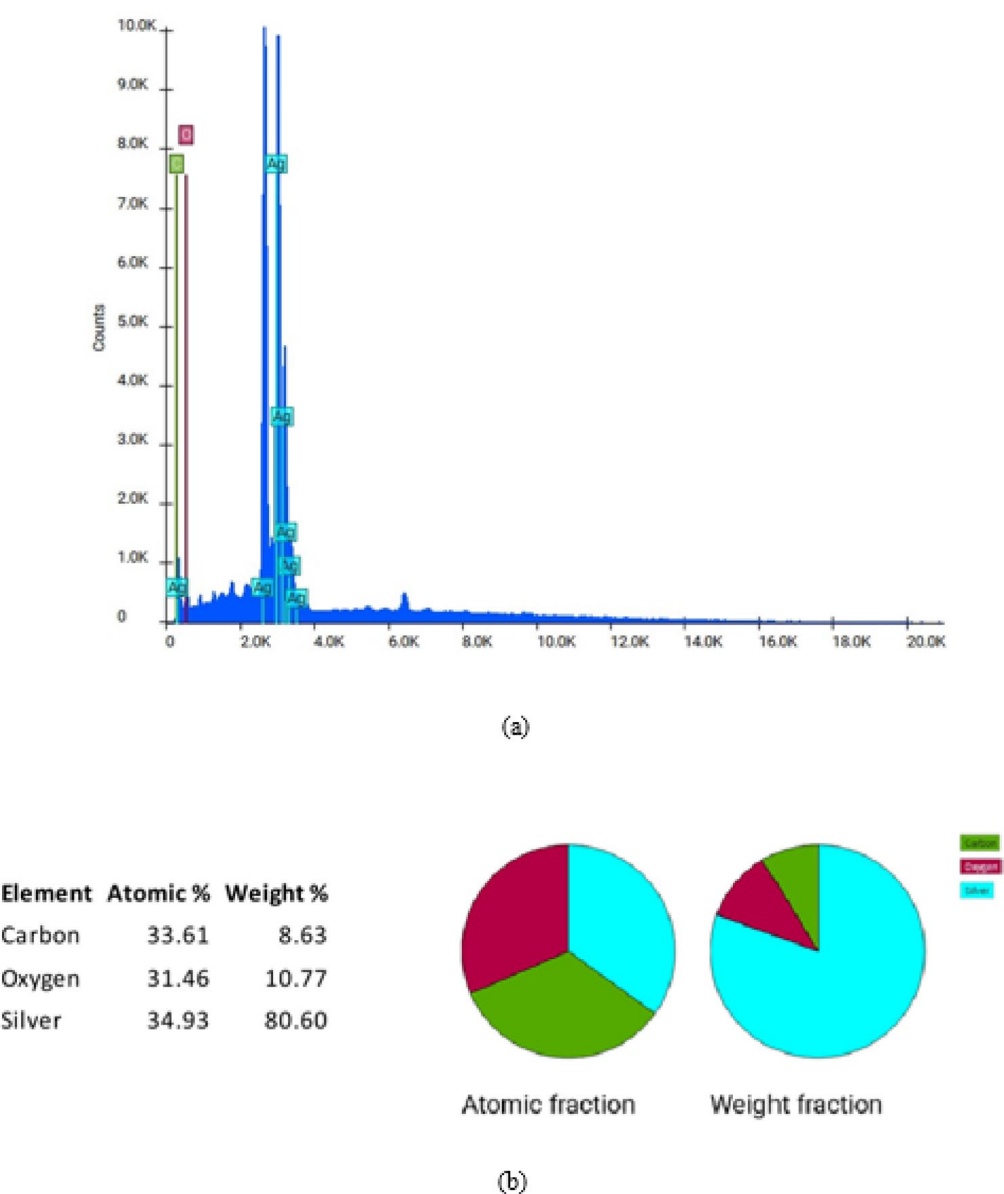
(**a**) EDX spectra of synthesised AgNPs from leaf extract of *B. pinnatum*. Weight proportion of synthesised AgNPs from leaf extract of *B. pinnatum*.

Analysis of the AgNPs attribute diffraction peaks in Fig. 2 along with miller indices with maximum peak at 38.25 degrees. Diffraction spectra is analyzed for confirmation with Crystallographis open data base (COD FILE in supplementary file 1). Synthesized AgNPs correspond to space group Imma (74) corresponding to orthorhombic with lattice parameters a = 4.75300 Å b = 7.48800 Å c = 8.16400 Å. Analysis of the SEM images revealed a higher density of nanoparticles at 500 nm and 2 μm, clearly indicating that the leaf extract assisted in the formation of silver nanoparticles with spherical geometry (Fig. 3). Morphological studies attribute bioorganic capping^37^of molecules is so high between the AgNPs that they interact via Hydrogen bonding and other electrostatic interactions. Existence of elements in the syntheiszed silver nanoparticles is analyzed with EDX spectra illustrated in Fig. 4(a) with appropriate weight fractions in Fig. 4(b).

**Fig. 5:**
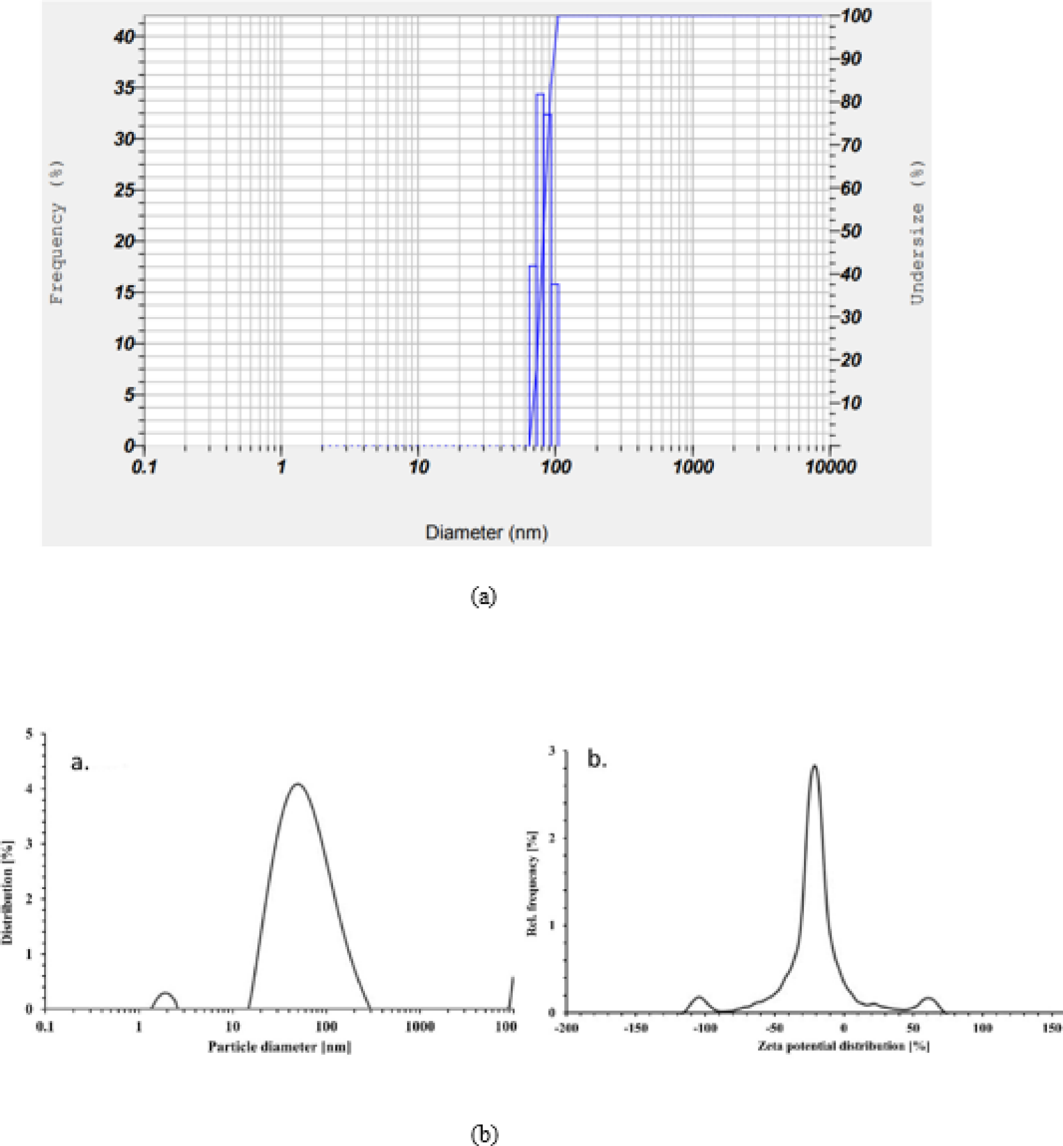
(**a**) Particle size distribution synthesised AgNPs from leaf extract of *B. pinnatum*. (**b**) Zeta potential distribution synthesised AgNPs from leaf extract of *B. pinnatum*.

An appropriate method for characterization of nanoparticles is identification of particles and its surface charge i via DLS and zeta potential Particle size distribution is illustrated in Fig. 5(a) with approximate size of 92 nm and with potential − 28.5mV in Fig. 5(b). Nanoparticles essentially connect with externally or internally located macromolecules with their surface charge. Interaction of nanoparticles with plant extract is influenced with changes in zeta potential and its distribution. Low value of Zeta potential at -25.8 mV^38^attributes stabilization of nanoparticles indicating greater probability with stability.

Investigative studies of particle distribution of AgNPs in an aqueous solution was determined using DLS technique. With polydispersive index (PI) of 0.211, is clear indication that the synthesized AgNPs are homogenized and spherical with particle size distributed around 92 nm as illustrated in Fig. 5(b). The findings are supported by research studies published on the particle size and charge of AgNPs^39^. The negative charge of synthesized AgNPs suggests that they can interact with biological macromolecules, as the interaction between NPs and cells dramatically depends on the NPs’ size. Previous studies have demonstrated that particles with a size of 100 nm have promising applications in both biomedical and agricultural fields^40^. The observed SPR peak at 430–450 nm not only confirmed the synthesis of AgNPs but also suggested particle sizes within the nanometer range, as longer wavelengths are indicative of larger particles—a result further validated by SEM and DLS analysis.

**Fig. 6:**
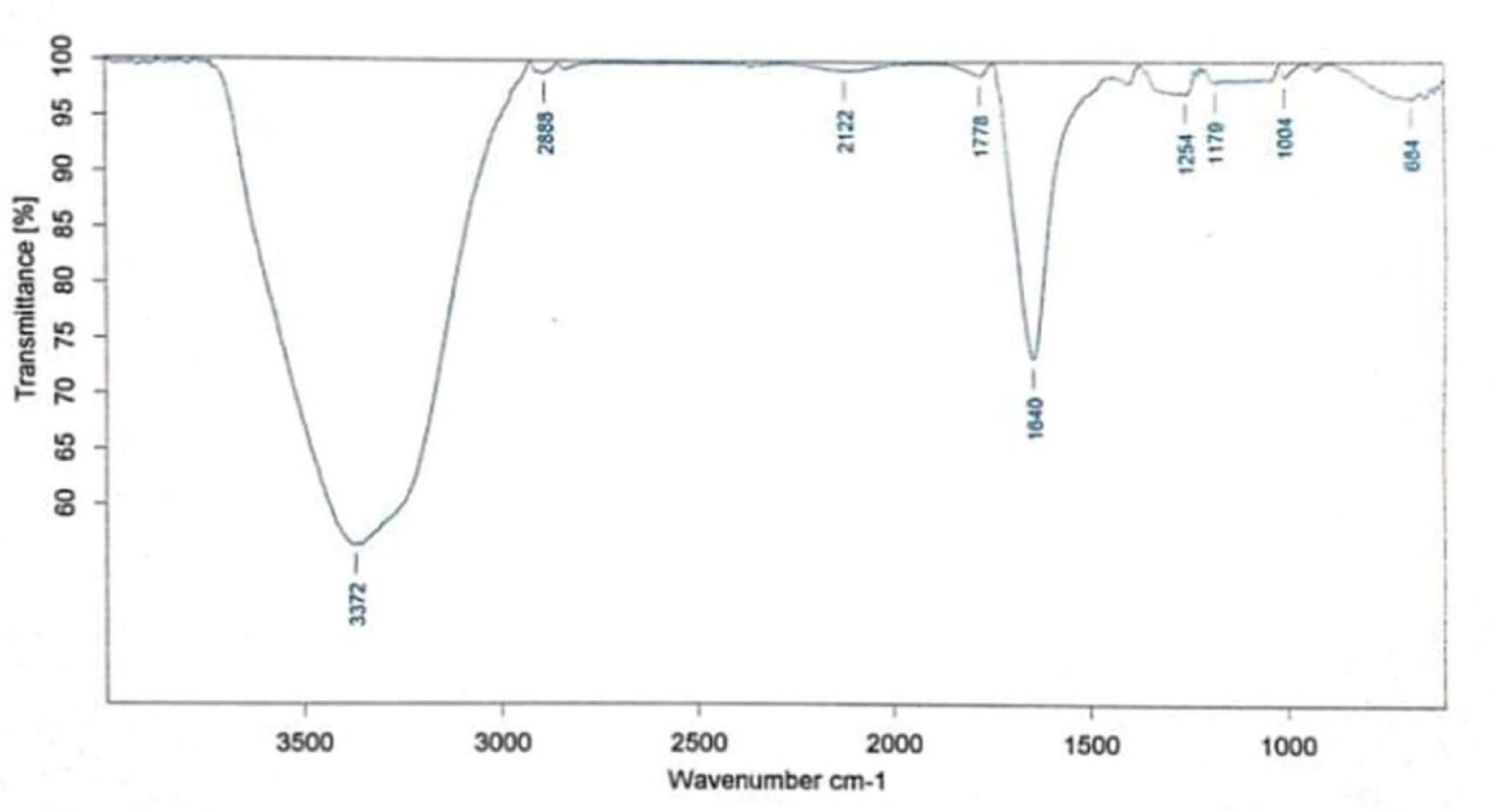
FTIR spectra of synthesised AgNPs from leaf extract of *B. pinnatum*.

FTIR analysis of AgNPs dispersed in distilled water revealed the presence of certain bond stretching and bending vibrations^37^including OH- stretching (3300 cm^− 1^), C-H stretching (2800 cm^− 1^), C = N– stretching (2100 cm^− 1^), C = O stretching (1770 cm^− 1^), NH- stretching (1640 cm^− 1^), O-H bending (1300 cm^− 1^), C = C bending (1000 cm^− 1^) and C = C bending (664 cm^− 1^) as shown in Fig. 6. Bond stretching and bending for OH-, C-O illustrates the participation of secondary metabolites such as tannins, saponins, and flavonoids. This finding is consistent with previous investigations that have shown that stretching of the OH bond affects the production of AgNPs^41^. Similarly, C = N, C = O, and N-H stretches revealed the presence of peptides or aromatic compounds with nitrogen, such as aniline. Tahir et al.^37^and Mamatha et al.^38^discovered a similar role for phytochemicals and C-H stretching, with C = C bending representing the presence of alkanes and alkenes in mono- and disubstituted forms.

### Effect of radiation/energy absorption

Response of radiation towards *B. pinnatum* extract and synthesized AgNPs exhibited changes in physical and chemical properties, including density, molecular weight, and chemical formula (atomic fraction and mole fraction) using Phy-X/PSD^43^(Table 2). Exposure of radiation influences physico-chemical effects of soil^41^with bioavailability^42^and particularly silver nanoparticles^43^influences to greater extent in EBF and EABF^44,45^. In this regard the plant extract and synthesized AgNPs were attributed for determining energy absorbing parameters with two different types of radiation.

**Table 2:** Physical and chemical properties of *B. pinnatum* extract and synthesized silver nanoparticles.

Name of compound	Density g/cm^3^	AMW g/mol	Formula
*B. pinnatum* extract	379,835	1695	C78H86O42
Synthesized silver nanoparticles	42,230	1863	C78H86O42 + AgNO3

Weight fraction of elements (%)		Mole fraction of elements (%)	
*B. pinnatum* extract	C	0.5525	0.3786
	H	0.0511	0.4175
	O	0.3963	0.2039
Synthesized silver nanoparticles	C	0.0528	0.3732
	H	0.0454	0.4019
	O	0.3864	0.2153
	Ag	0.0579	0.0048
	N	0.0075	0.0048

**Fig. 7:**
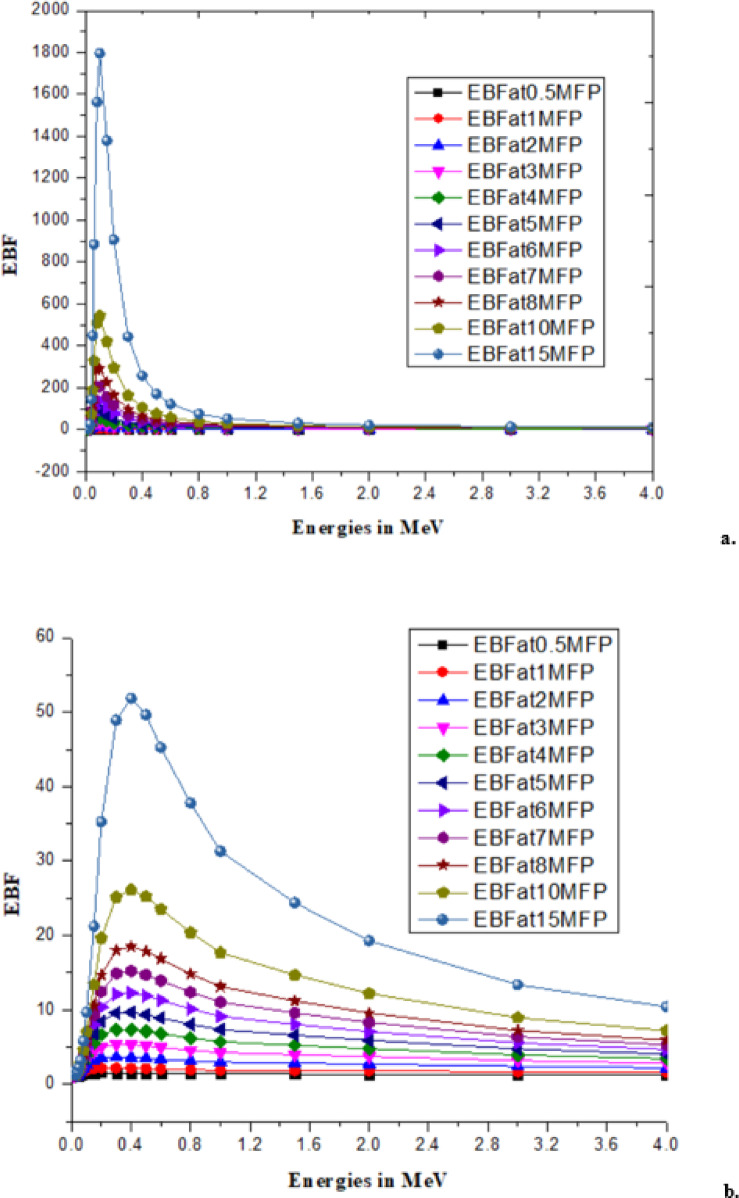
EBF of (**a**) *B. pinnatum* extract with Rubydium and (**b**) Synthesized Silver Nanoparticles with Rubydium.

**Fig. 8:**
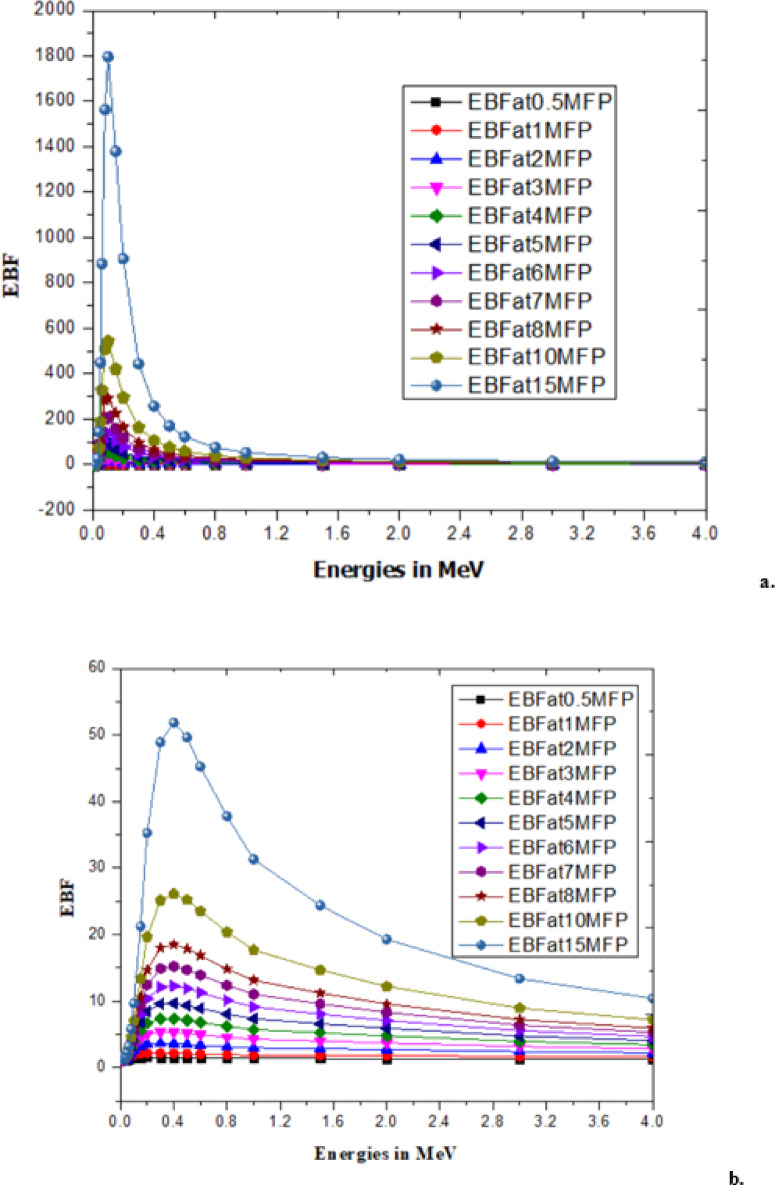
EBF of (**a**) *B. pinnatum* extract with cesium and (**b**) synthesized silver nanoparticles with cesium.

**Fig. 9:**
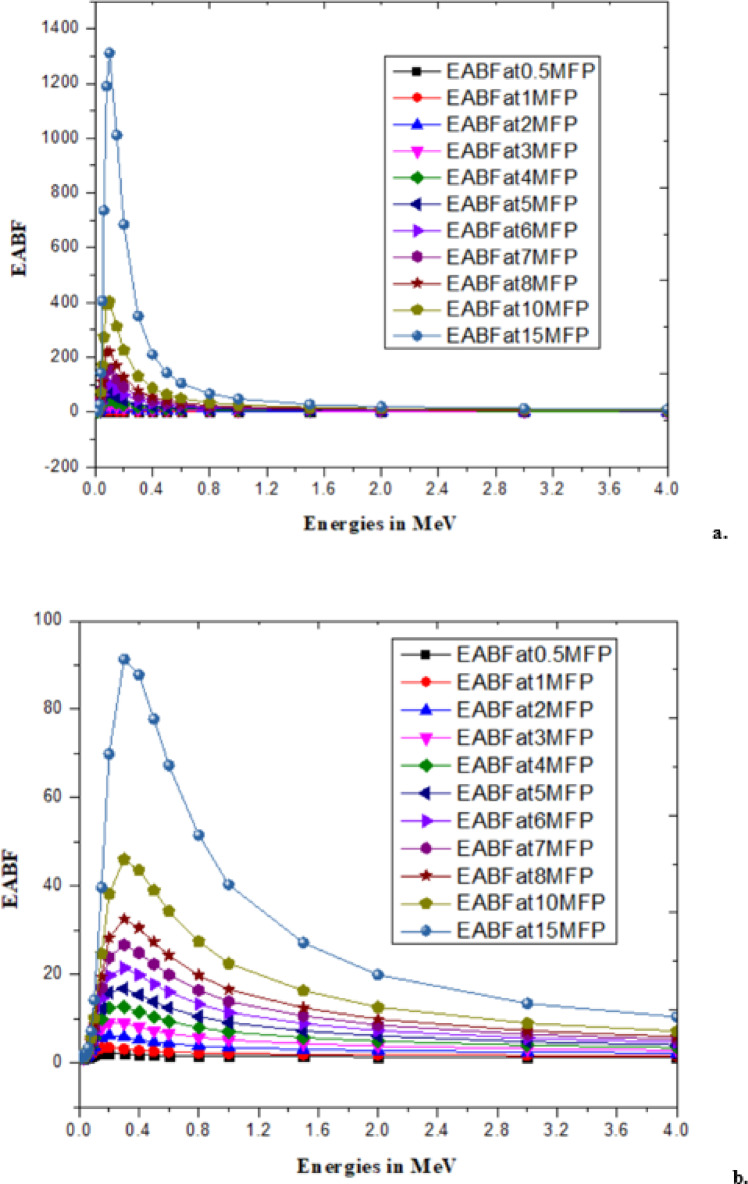
EABF of (**a**) *B. pinnatum* extract with Rubydium and (**b**) EABF of Synthesized Silver Nanoparticles with Rubydium.

**Fig. 10:**
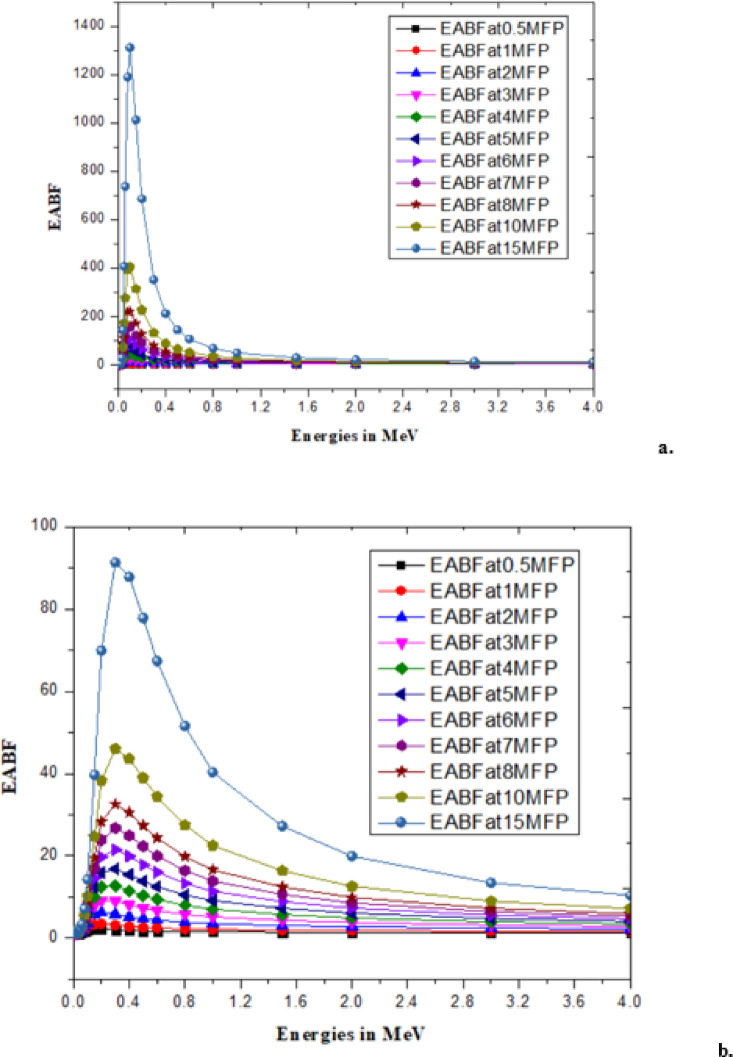
EABF of (**a**) *B. pinnatum* extract with cesium and (**b**) synthesized silver nanoparticles with cesium.

With low energy radiation (Rubydium, Rb)^46,47^and high energy radiation (Cesium, Cs)^47^has significantly contributed to changes in the *B. pinnatum* extract and the synthesized AgNPs which are observed as radiation parameters like linear attenuation coefficient and mass attenuation coefficient^48^, effective atomic number and effective electron density^49^as shown in the Table 3. An illustration of EBF and EABF due to the mean free path (MFP) at different energies for Rb and Cs is shown in Figs. 7, 8, 9 and 10.

**Table 3:** Response of radiation to *B. pinnatum* extract and synthesized silver nanoparticles.

Properties	With Rb in counts/sec			With Cs in counts/sec		
	B. pinnatum extract	Silver nitrate	Synthesized silver nanoparticles	B. pinnatum extract	Silver nitrate	Synthesized silver nanoparticles
Density (g/cm^3^)	379,835	4718	42,230	379,835	4718	42,230
AMW	379,835	4718	42,230	379,835	4718	42,230
MAC	1	23	3	0	6040	3322
LAC	611,397	167,461	145,028	0	2.7 × 10^7^	1.4 × 10^8^
ECS	3.1 × 10^− 23^	4.7 × 10^− 23^	3.2 × 10^− 23^	2.54 × 10^− 24^	3.4 × 10^− 23^	3.4 × 10^− 24^
Zeff	6.7	45.7	23.5	6.6	45.5	23.7
Neff	4.805 × 10^23^	8.1 × 10^23^	1.6 × 10^24^	4.7 × 10^23^	8 × 10^23^	1.6 × 10^24^

The changes (process of metabolism) are mechanized with the interaction of radiation with matter that influences ionization, and electron transfer is explored with the responsible parameters EBF and EABF^46,49^. Interaction of mean free path and EBF values are tabulated in supplementary material (File 2) for the response of EBF with Rb (Beta radiation) and Cs (Gamma radiation) with the theoretically predicted values using G-P fitting parameters^46,47^and illustrated upto15MFP for clear identification of EBF Fig. 7(a, b) due to Rb and EBF in Fig. 8(a, b) for Cs with *B. pinnatum* extract and its synthesized AgNPs. Corresponding to the response of EABF theoretically predicted with G-P fitting parameters (Typical G-P fitting parameters of EABF with Rb in supplementary material) are illustrated in Fig. 9(a, b) with Rb (Beta radiation) and Fig. 10(a, b) with Cs (Gamma radiation).

Studies attribute that molecular interaction is vital that influenced the reduction in parameters with increasing energies with low beta (Rb) and high gamma radiation (Cs). In conclusion, the dominant role of metal in the synthesis of nanoparticles with greater soil withstand capacity, even during prolonged exposure to high-energy radiation, makes them useful for biological studies.

### Determination of growth, biochemical, and agronomical parameters

The growth parameters, such as G%, SL, RL, and SVI, are examined to assess the growth characteristics of rice seedlings, and the results are summarized in Table 4. G%, SL, and RL all steadily increased in control and under F-stress conditions in seeds primed with AgNPs (50 mg/l) and fertilizer. Variation was observed in G%, SL, and RL under F-stressed conditions. Plants treated with AgNPs exhibited less withering and rolling leaves than controls in F soils in 2 weeks. The results indicate that treatment with NPs positively influenced G% and shoot and root growth in the plants over time. The probable mechanism is that NPs induce enzymes such as α-amylase and proteases, thereby increasing the total protein content in germinating seeds and promoting water uptake^50,51^. Our results were supported by Tahir et al.^37^and Sultana et al.^9^, which showed that the relative water content was considerably improved in AgNPs-treated groups compared to the controls in rice plants.

**Table 4:** Rice’s morphological traits in soils with fertilizer and NPs spray in both normal and F-rich conditions. Plants treated with fertilizer in normal (N-F) and fluoride rich soils (F-F) and AgNPs treated in normal (N-AgNPs) and fluoride rich soils (F- AgNPs).

Morphological characteristics	Normal Soil						Fluoride rich soil					
	Control (*N*-F)			Ag NPs (*N*-Ag)			Control (F-F)			Ag NPs (F-Ag)		
Days	D-1	D-15	D-30	D-1	D-15	D-30	D-1	D-15	D-30	D-1	D-15	D-30
Germination %	90.0 ± 1.02	98.0 ± 1.40	100 ± 1.74	92.0 ± 1.20	99.0 ± 1.50	100 ± 0.4	85 ± 1.15	94.0 ± 1.52	98.0 ± 1.33	89.0 ± 1.6	96.0 ± 1.2	99.0 ± 1.15
Shoot length (cm)	0.18 ± 0.8	6.16 ± 0.10	7.81 ± 0.12	0.25 ± 0.11	7.43 ± 0.13	8.16 ± 0.10	0.11 ± 0.10	5.15 ± 0.09	6.56 ± 0.13	0.12 ± 0.08	6.22 ± 0.09	7.63 ± 0.13
Root length (cm)	0.98 ± 0.9	6.30 ± 0.11	8.21 ± 1.45	1.65 ± 0.09	6.90 ± 0.11	8.76 ± 0.12	0.76 ± 0.09	4.53 ± 0.17	5.83 ± 0.18	1.02 ± 0.09	5.42 ± 0.16	6.88 ± 0.20
Vigour index	103.4± 2.4	1224.08± 14.3	1601.04± 18.64	173.64± 1.88	1417.57± 15.67	1693.05± 18.33	73.35± 1.24	909.1± 12.48	1215.88± 14.56	101.68 ± 1.05	1117.12± 11.67	1436.37± 16.42

**Fig. 11:**
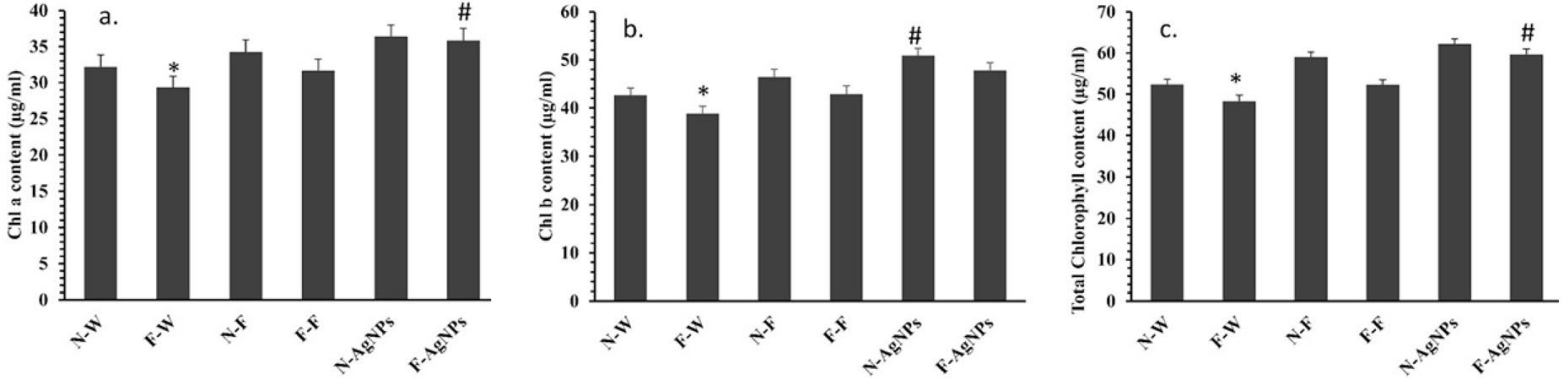
Effect of different treatments of water, foliar fertilizer and AgNPs on the chlorophyll content of rice plants in both normal and F rich soils. (**a**) Chlorophyll a, (**b**) Chlorophyll b, (**c**) Total Chlorophyll content. Plants treated with water in normal (N-W) and fluoride rich soils (F-W), fertilizer treated in normal (N-F) and fluoride rich soils (F-F) and AgNPs treated in normal (N-AgNPs) and fluoride rich soils (F- AgNPs). The data are the mean ± SD (*n* = 10), The asterisk denotes that data is statistically significant from N-W, whereas the hash mark indicates that data are significantly different from F-W (analysed by ANOVA; *p* ≤ 0.05).

The biochemical parameters (chlorophyll, antioxidant enzymes, and phenols) assessed showed the influence of AgNPs on the plant physiology. Chlorophyll directly contributes to the metabolism of carbohydrates during photosynthesis as well as under stress conditions. There is increased ROS generation, a decrease in chlorophyll content, and increased lipid peroxidation, disrupting key elements of photosynthesis^52^. It has been established that stress reduces Chl levels in plants^53^. The current study observed a pronounced decrease in Chl a, b, and total Chl concentration in rice plants under F stress. In comparison to control in F-rich soils, it was observed that rice seeds treated with AgNPs had higher levels of Chl a (13.3%), Chl b (11.4%), and total Chl (14.0%) equal with fertilizer, as shown in Fig. 11. Compared to the control with rice seeds treated with AgNPs and fertilizer in normal soil, the increase in Chl a, Chl b, and total Chl content was observed. The present study demonstrated that treating seeds with AgNPs under F-stress conditions enhances chlorophyll content, thereby improving physiological processes.

**Fig. 12:**
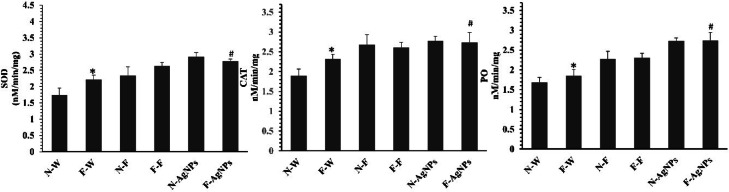
Effect of different treatments of water, foliar fertilizer and AgNPs on the enzymes of rice plants in both normal and F rich soils. (**a**) SOD, (**b**) CAT, (**c**) PO. Plants treated with water in normal (N-W) and fluoride rich soils (F-W), fertilizer treated in normal (N-F) and fluoride rich soils (F-F) and AgNPs treated in normal (N-AgNPs) and fluoride rich soils (F- AgNPs). The data are the mean ± SD (*n* = 10), The asterisk denotes that data is statistically significant from N-W, whereas the hash mark indicates that data are significantly different from F-W (analysed by ANOVA; *p* ≤ 0.05).

Plants often increase antioxidant enzyme activity to maintain ROS levels in plant cells^54^. The enzyme activities were determined in plants treated with AgNPs and in the control, under both normal and F-rich soils, to establish the possible involvement of AgNPs in ROS homeostasis. Since chloroplasts are the most vulnerable organelles to oxidative stress, F-rich soils cause plants to accumulate high quantities of oxidative stress molecules, halting plant development^13^. Increased ROS-scavenging activity, such as SOD, CAT, and PO, supports plant physiology. In this study, in both normal and fluoride-rich soils, the levels of these enzymes were lower in control plants when compared to the plant seeds primed with AgNPs and fertilizer in Fig. 12. Plants exposed to F-stress showed increased SOD activity (6%), which was significant Fig. 12 (a). PO and CAT activities have a direct relationship with SOD activity, illustrated in Fig. 12 (b, c). Compared to controls in F-rich soils, the CAT and PO activity in rice seeds treated with AgNPs (50 mg/l) rose by 5% and 19%, respectively. This study found that AgNPs priming reduced ROS accumulation under stress, thereby supporting increased F tolerance in rice via a ROS-detoxification mechanism. Numerous plant cell functions depend on the stable state of ROS accumulation, which is controlled by a delicate balance between scavenging enzymes and the free radicals they produce^39^. According to the present study, higher levels of SOD, CAT, and PO activity under F-stress conditions helped plants detoxify excess ROS and reduced ROS levels in AgNPs-primed plants. Previous research showed that AgNPs increased SOD, CAT, and PO levels and validated these findings^55^. An acclimatization response might have been triggered when ROS signaling modified the redox status of several regulatory proteins, altered transcription, and altered translation.

**Fig. 13:**
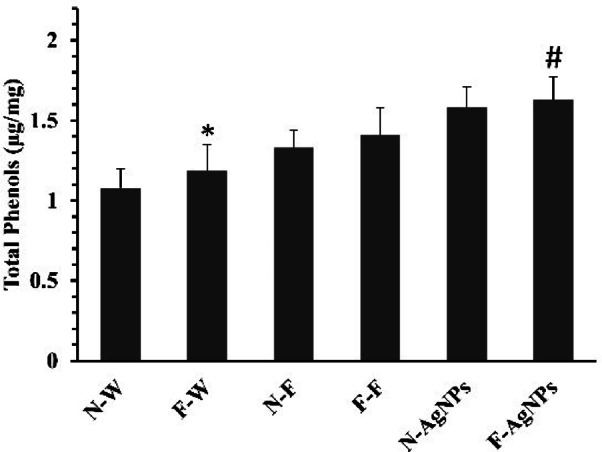
Effect of different treatments of water, foliar fertilizer and AgNPs on total phenolic content of rice plants in both normal and F rich soils. Plants treated with water in normal (N-W) and fluoride rich soils (F-W), fertilizer treated in normal (N-F) and fluoride rich soils (F-F) and AgNPs treated in normal (N-AgNPs) and fluoride rich soils (F- AgNPs). The data are the mean ± SD (*n* = 10), The asterisk denotes that data is statistically significant from N-W, whereas the hash mark indicates that data are significantly different from F-W (analysed by ANOVA; *p* ≤ 0.05).

The phenolic levels in AgNPs primed F-rich soils were slightly lower. The total phenolic content in the plant’s leaves treated with AgNPs (50 mg/l) increased by 15.6% and 18.7% in both F and normal soils, respectively, as shown in Fig. 13. Thus, according to the total phenolic content analysis, AgNP-treated plants showed a shift toward secondary metabolism, and AgNPs raised the amount of phenol present in the plant leaves. Higher phenolic derivatives reduce ROS and lipid alkoxy radicals by acting as metal chelators and antioxidants^9^. Accordingly, priming AgNPs decreased ROS levels by increasing their scavenging activity and detoxifying excess ROS, thereby protecting chlorophyll pigments and ultimately maintaining plant growth and development.

**Fig. 14:**
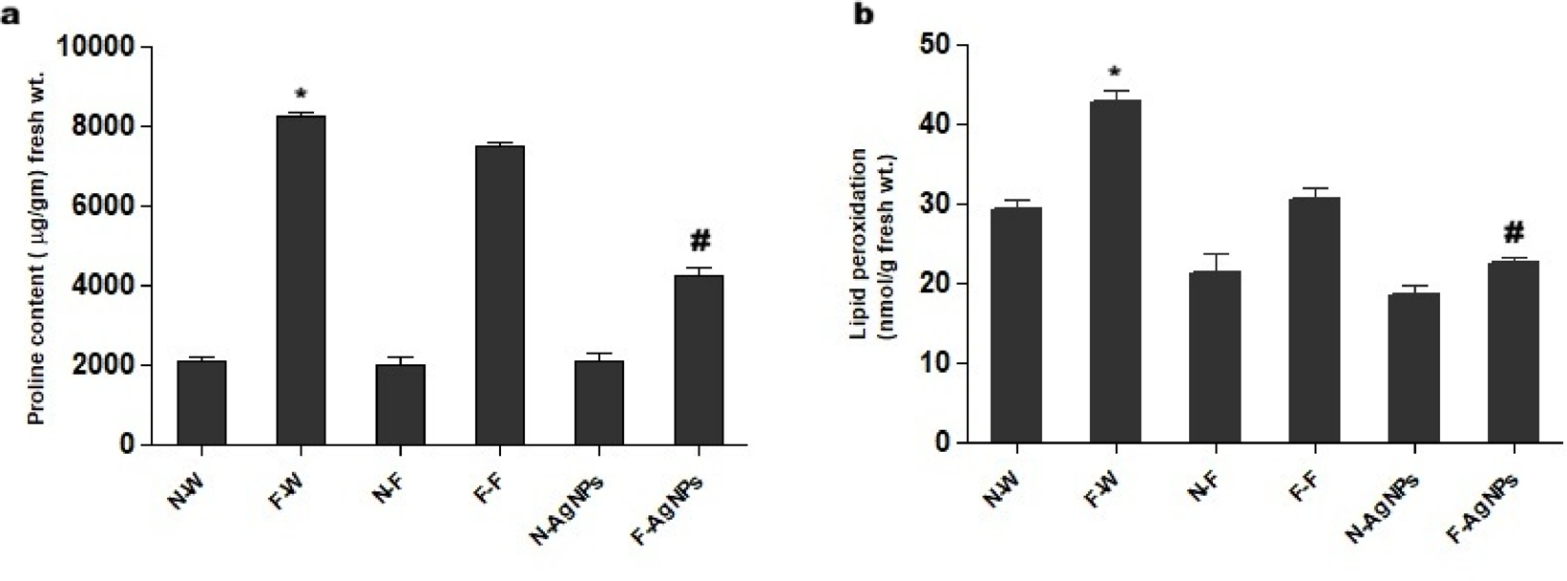
Effect of different treatments of water, foliar fertilizer and AgNPs on (**a**) proline and (**b**) MDA content of rice plants in both normal and F rich soils. Plants treated with water in normal (N-W) and fluoride rich soils (F-W), fertilizer treated in normal (N-F) and fluoride rich soils (F-F) and AgNPs treated in normal (N-AgNPs) and fluoride rich soils (F- AgNPs). The data are the mean ± SD (*n* = 10), The asterisk denotes that data is statistically significant from N-W, whereas the hash mark indicates that data are significantly different from F-W (analysed by ANOVA; *p* ≤ 0.05).

Proline functions as a reactive oxygen species (ROS) scavenger and maintains redox homeostasis and protects macromolecules during stress conditions. Proline increases under fluoride (abiotic) stress as a protective response. The proline content in AgNPs primed F-rich soils reduced oxidative stress and decrease proline content reflecting a mitigated stress reaction (Fig. 14a). Although proline levels in F-AgNPs remained higher than the normal control, the reduction suggests that AgNP application alleviated fluoride-induced stress, thereby lowering the need for excessive osmoprotectant synthesis. This indicates an improved physiological status of plants due to nanoparticle-mediated stress mitigation. The malonaldehyde (MDA) levels increased significantly in F-rich soils reflecting enhanced membrane damage caused by oxidative stress (Fig. 14b). Plants treated with AgNPs significantly decreased MDA levels when grown in F-rich soils demonstrating a protective effect against oxidative damage. The reduced lipid peroxidation suggests that AgNPs enhanced the antioxidant defense system of rice plants, either by directly scavenging ROS or by stimulating endogenous antioxidant enzymes. These enhanced antioxidant enzyme responses, increased phenolic accumulation, decreased proline and MDA content suggest a potential shift towards osmo-protective metabolism.

**Fig. 15:**
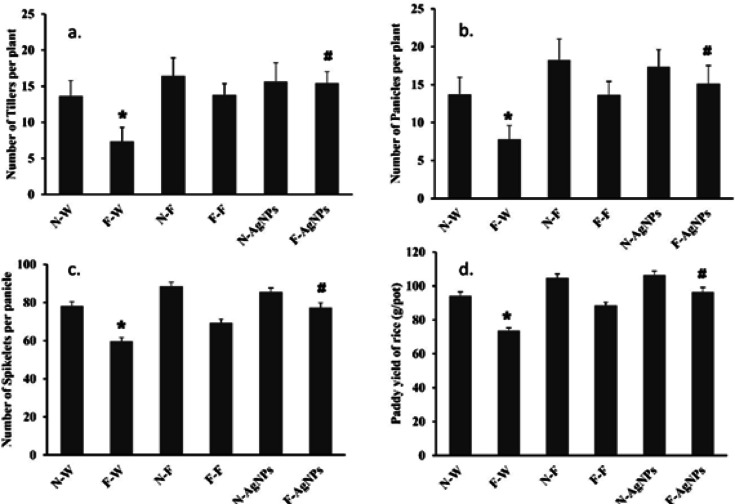
Effect of different treatments of water, foliar fertilizer and AgNPs on (**a**) Total number of Tillers, (**b**) Panicles, (**c**) Spikelets, (**d**) Rice yield g per pot. Plants treated with water in normal (N-W) and fluoride rich soils (F-W), fertilizer treated in normal (N-F) and fluoride rich soils (F-F) and AgNPs treated in normal (N-AgNPs) and fluoride rich soils (F- AgNPs). The data are the mean ± SD (*n* = 10), The asterisk denotes that data is statistically significant from N-W, whereas the hash mark indicates that data are significantly different from F-W (analysed by ANOVA; *p* ≤ 0.05).

**Fig. 16:**
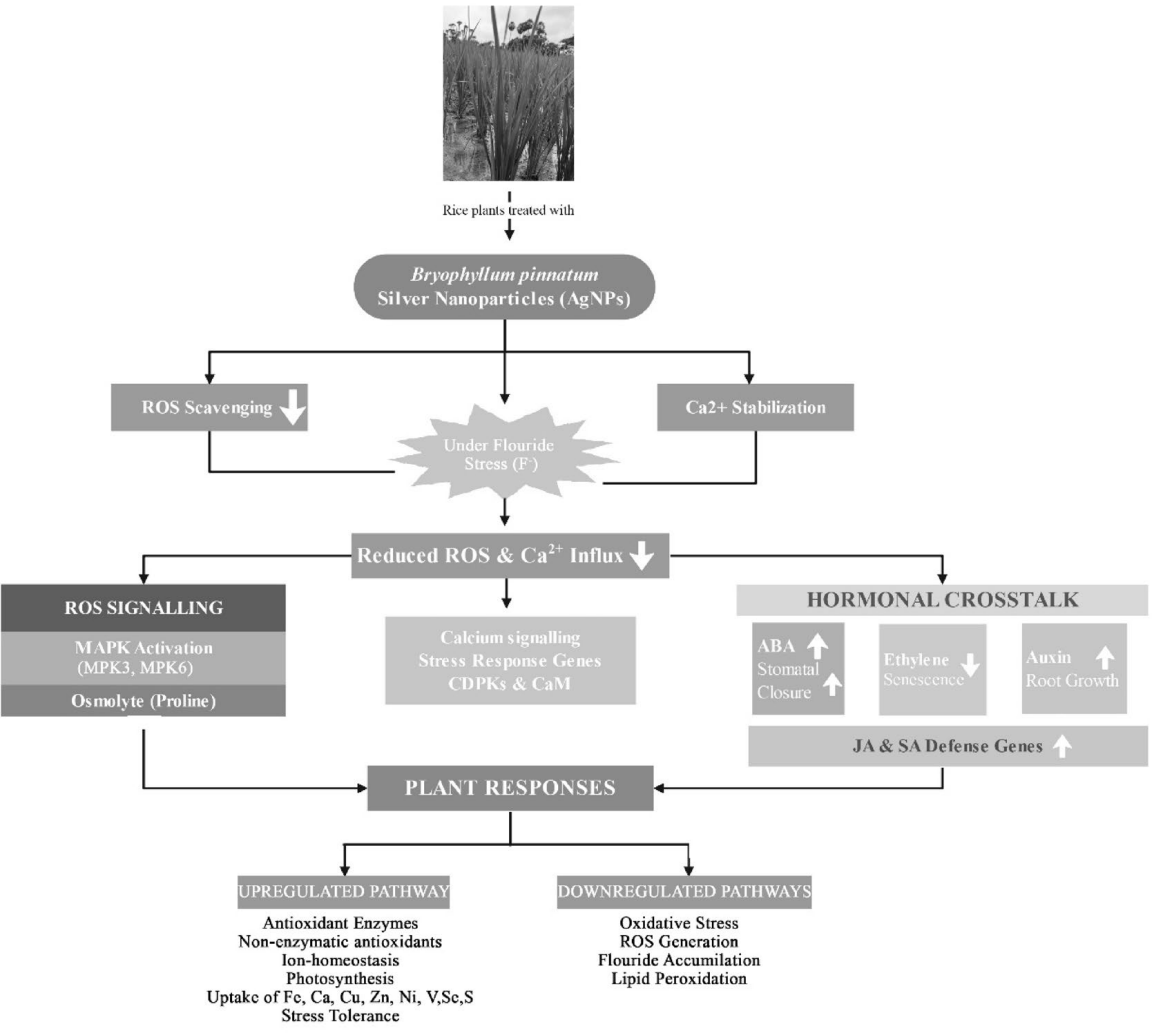
Proposed molecular and physiological mechanisms of *B. pinnatum* silver nanoparticles in alleviating fluoride stress in rice plants.

Silver is reported to be a growth simulator, and AgNPs can enhance plant metabolism, thereby increasing yield^56,57^. The agronomic profile indicates a plant’s optimal homeostasis and physiological wellness, producing a good yield which can be generated from the characteristics such as number of tillers per plant, number of panicles per plant, number of spikelets per panicle, and rice yield per pot Fig. 15. The information explicitly shows that AgNPs priming significantly increased the number of tillers (11.74%), panicles (10.75%), spikelets (11.32%), and yield per pot (8.97%) when compared to controls Fig. 15. According to Tahir et al.^37^and Sultana et al.^9^, priming with AgNPs in rice improved biochemical homeostasis in plants, enhancing plant growth and yield. Similarly, in mung bean^58^, wheat^59^, and fenugreek^60^, treatment with AgNPs improved plant growth and yield. Waqas Mazhar et al.‘s^10^experiments supported our results, demonstrating that nano-priming can increase rice tillers, panicles, and spikelets, as well as the nutritional profile of rice. All these studies report that NPs increase the synthesis of enzymes involved in nutrient uptake and acquisition, which is probably due to increased photosynthetic pigments, ROS-scavenging activity, and photosynthetic rate. This study indicates that AgNPs priming regulates the fluoride response by preserving photosynthetic pigments and repressing ROS accumulation, thereby improving rice yield (Fig. 16).

## Conclusion

The present study assessed the physical, chemical, and microbiological properties of F-stress soil to select a suitable crop, understand the microbial flora, and optimize yield. AgNPs were synthesized via a green synthesis approach, and biophysical techniques revealed the morphology of the AgNPs. High-energy studies on the B. pinnatum plant extract and AgNPs demonstrated that the amount of energy absorbed by the plant extract is greater when exposed to radiation than that of AgNPs. These studies demonstrate the stability of AgNPs, making them a suitable and stable molecule for application in these fields. Thus, the present study examined AgNPs to mitigate F stress, and they positively impacted plant growth in both normal and F-rich soils. AgNPs significantly regulate ROS levels by enhancing photosynthetic pigments, ROS-scavenging activity, and yield. The research suggests a potential role for AgNPs in regulating F-sensitive plant defense systems to develop F tolerance in plants for sustainable agriculture.

## Supplementary Information

Below is the link to the electronic supplementary material.

Supplementary Material 1

Supplementary Material 2

## Data Availability

The experiment supports the findings of this study and is available in the supplementary material.
