# Supplementary material for: Influence of radiation on green-synthesized AgNPs and their role in enhancing fluoride stress tolerance in rice
Source: Sci Rep. 2026 Mar 1;16:11503. doi: 10.1038/s41598-026-40077-6 (PMC13057137; doi:10.1038/s41598-026-40077-6)
Supplement: Supplementary file 2 — Supplementary Material 2 [file 41598_2026_40077_MOESM2_ESM.pdf]

Entry # 96-150-9794

Phase classification

**Name**	
**Formula**	Ag_2Eu
**I/Ic**	7.960000
**Sample Name**	1509793
**Quality**	C (calculated)

References

Publication

**Bibliography**	Koester W., Meixner J., "Der Aufbau der Systeme des Europiums mit Silber, Kadmium und Indium sowiedas System Kadmium-Strontium", Zeitschrift fuer Metallkunde **56**, 695-703 (1965).

Origin of data

**Source of entry**	COD (Crystallography Open Database)
**Link to orig. entry**	1509793

Crystal structure

Crystallographic data

**Space group**	I m m a (74)
**Crystal system**	orthorhombic
**Cell parameters**	a= 4.75300 Å b= 7.48800 Å c= 8.16400 Å
**Z**	4

Atom coordinates

Element	Oxid.	x	y	z	Bi	Focc
Ag		0.000	0.560	0.330	1.000000	1.000000
Eu		0.000	0.250	0.040	1.000000	1.000000

Diffraction data

Diffraction lines

d [Å]	Int.	h	k	l	Mult.
5.5184	70.8	0	1	1	4
4.1076	38.3	1	0	1	4
4.0820	1.7	0	0	2	2
3.7440	2.1	0	2	0	2
2.8617	440.4	1	1	2	8
2.7670	1000.0	1	2	1	8
2.7592	473.6	0	2	2	4
2.5577	93.1	0	1	3	4
2.3869	293.0	0	3	1	4
2.3765	441.2	2	0	0	2
2.3616	683.9	1	0	3	4
2.1827	11.0	2	1	1	8
2.0538	0.9	2	0	2	4
2.0410	0.3	0	0	4	2
2.0064	0.3	2	2	0	4
1.9975	17.9	1	2	3	8
1.9433	59.6	1	3	2	8
1.8720	39.9	0	4	0	2
1.8395	22.9	0	3	3	4
1.8192	16.0	1	1	4	8
1.8007	242.5	2	2	2	8
1.7920	55.6	0	2	4	4
1.7410	53.5	2	1	3	8
1.7034	82.1	1	4	1	8
1.7016	32.7	0	4	2	4
1.6841	177.0	2	3	1	8
1.5953	71.4	0	1	5	4
1.5553	2.7	3	0	1	4
1.5484	0.2	2	0	4	4
1.5442	9.3	1	0	5	4
1.4993	240.6	1	3	4	8
1.4730	24.4	0	5	1	4
1.4706	32.8	2	4	0	4
1.4670	36.6	1	4	3	8

1.4546	19.3	2	3	3	8
1.4491	44.5	3	1	2	8
1.4363	108.7	3	2	1	8
1.4308	47.4	2	2	4	8
1.4276	41.5	1	2	5	8
1.3835	30.0	2	4	2	8
1.3796	5.4	0	4	4	4
1.3692	96.1	3	0	3	4
1.3664	0.1	0	3	5	4
1.3607	22.0	0	0	6	2
1.3482	103.3	1	5	2	8
1.3245	69.4	2	1	5	8
1.3120	10.5	0	5	3	4
1.2886	37.5	1	1	6	8
1.2859	2.9	3	2	3	8
1.2788	14.7	0	2	6	4
1.2711	11.1	3	3	2	8
1.2520	25.4	2	5	1	8
1.2480	26.4	0	6	0	2
1.2344	4.0	3	1	4	8
1.1963	21.6	3	4	1	8
1.1941	7.2	1	6	1	8
1.1935	2.0	0	6	2	4
1.1931	6.2	2	4	4	8
1.1912	1.6	1	4	5	8
1.1882	35.4	4	0	0	2
1.1846	0.1	2	3	5	8
1.1808	25.3	2	0	6	4
1.1703	3.0	1	5	4	8
1.1616	0.9	4	1	1	8
1.1586	15.8	1	3	6	8
1.1524	2.8	0	1	7	4
1.1486	12.9	2	5	3	8
1.1409	0.2	4	0	2	4
1.1370	2.8	3	0	5	4
1.1327	5.1	1	0	7	4
1.1326	0.0	4	2	0	4
1.1261	18.3	2	2	6	8
1.1188	78.9	3	3	4	8
1.1051	12.8	3	4	3	8
1.1049	34.3	2	6	0	4
1.1037	39.2	0	5	5	4
1.1034	50.0	1	6	3	8
1.1006	0.2	0	4	6	4
1.0913	37.3	4	2	2	8
1.0880	15.1	3	2	5	8
1.0842	0.6	1	2	7	8
1.0776	9.2	4	1	3	8
1.0665	2.8	2	6	2	8
1.0647	0.2	0	6	4	4
1.0637	31.5	4	3	1	8
1.0606	6.0	0	7	1	4
1.0566	39.2	0	3	7	4
1.0516	42.7	3	5	2	8
1.0369	4.1	2	1	7	8
1.0269	0.0	4	0	4	4
1.0226	17.6	3	1	6	8
1.0205	6.7	0	0	8	2
1.0111	0.1	1	7	2	8
1.0032	8.8	4	4	0	4
1.0010	60.9	2	5	5	8
0.9987	0.3	2	4	6	8
0.9981	5.4	4	3	3	8
0.9956	3.0	0	7	3	4
0.9903	13.7	4	2	4	8
0.9890	24.6	1	1	8	8
0.9852	19.6	1	5	6	8
0.9846	0.4	0	2	8	4
0.9742	9.8	4	4	2	8
0.9734	4.2	3	6	1	8
0.9718	0.9	3	4	5	8
0.9717	0.4	2	6	4	8
0.9706	0.6	1	6	5	8
0.9691	0.7	1	4	7	8

0.9686	9.9	2	7	1	8
0.9655	65.2	2	3	7	8
0.9603	1.7	3	5	4	8
0.9539	9.8	3	3	6	8
0.9530	25.9	4	1	5	8
0.9442	0.6	5	0	1	4
0.9392	3.4	3	0	7	4
0.9377	11.8	2	0	8	4
0.9360	0.5	0	8	0	2
0.9292	28.7	1	7	4	8
0.9265	0.1	1	3	8	8
0.9248	11.3	4	5	1	8
0.9223	35.4	3	6	3	8
0.9202	0.4	0	5	7	4
0.9197	6.0	0	6	6	4
0.9188	11.4	5	1	2	8
0.9182	5.6	2	7	3	8
0.9156	28.9	5	2	1	8
0.9126	36.3	1	8	1	8
0.9123	17.7	0	8	2	4
0.9110	0.5	3	2	7	8
0.9096	0.7	2	2	8	8
0.9005	5.1	0	1	9	4
0.9003	3.4	4	4	4	8
0.8974	30.7	5	0	3	4
0.8966	0.1	4	3	5	8
0.8960	1.6	0	4	8	4
0.8950	14.3	4	0	6	4
0.8948	1.1	0	7	5	4
0.8910	3.7	1	0	9	4
0.8807	8.2	4	5	3	8
0.8727	1.1	5	2	3	8
0.8709	1.0	2	8	0	4
0.8705	12.6	4	2	6	8
0.8701	6.0	1	8	3	8
0.8680	4.8	5	3	2	8
0.8668	38.1	1	2	9	8
0.8664	0.1	3	7	2	8
0.8606	25.8	4	6	0	4
0.8581	0.9	2	5	7	8
0.8577	13.0	2	6	6	8
0.8561	2.1	5	1	4	8
0.8526	2.1	0	3	9	4
0.8524	25.0	3	1	8	8
0.8517	38.8	2	8	2	8
0.8508	9.4	0	8	4	4
0.8499	20.2	3	5	6	8
0.8431	13.1	5	4	1	8
0.8421	11.5	2	1	9	8
0.8421	2.4	4	6	2	8
0.8405	0.7	3	6	5	8
0.8395	0.7	3	4	7	8
0.8387	4.1	1	6	7	8
0.8384	3.6	2	4	8	8
0.8374	2.6	2	7	5	8
0.8304	60.5	1	5	8	8
0.8281	13.7	1	7	6	8
0.8277	2.5	0	9	1	4
0.8273	4.2	4	1	7	8
0.8215	2.3	5	0	5	4
0.8164	6.9	0	0	10	2
0.8145	72.1	5	3	4	8
0.8132	39.0	3	7	4	8
0.8113	0.1	3	3	8	8
0.8092	12.8	5	4	3	8
0.8087	79.2	4	5	5	8
0.8075	0.4	4	4	6	8
0.8045	6.1	1	4	9	8
0.8035	0.7	1	9	2	8
0.8025	5.9	2	3	9	8
0.8024	17.2	5	2	5	8
0.8020	56.0	3	8	1	8
0.8010	26.9	2	8	4	8
0.8004	27.1	1	8	5	8

0.8000	0.2	1	1	10	8
0.7977	2.7	0	2	10	4
0.7956	5.0	0	9	3	4
0.7930	0.6	4	6	4	8
0.7922	35.5	6	0	0	2
0.7913	17.8	4	7	1	8
0.7900	12.8	0	6	8	4
0.7896	122.0	4	3	7	8
0.7883	34.9	0	7	7	4
0.7875	71.3	5	5	2	8
0.7872	7.7	3	0	9	4
0.7841	1.3	6	1	1	8
0.7817	10.4	2	9	1	8
0.7777	0.3	6	0	2	4
0.7759	25.5	0	5	9	4
0.7751	60.2	5	1	6	8
0.7750	0.0	6	2	0	4
0.7742	52.7	4	0	8	4
0.7727	36.0	3	8	3	8
0.7721	77.0	2	0	10	4
0.7712	199.6	0	8	6	4

Diffraction pattern graphics

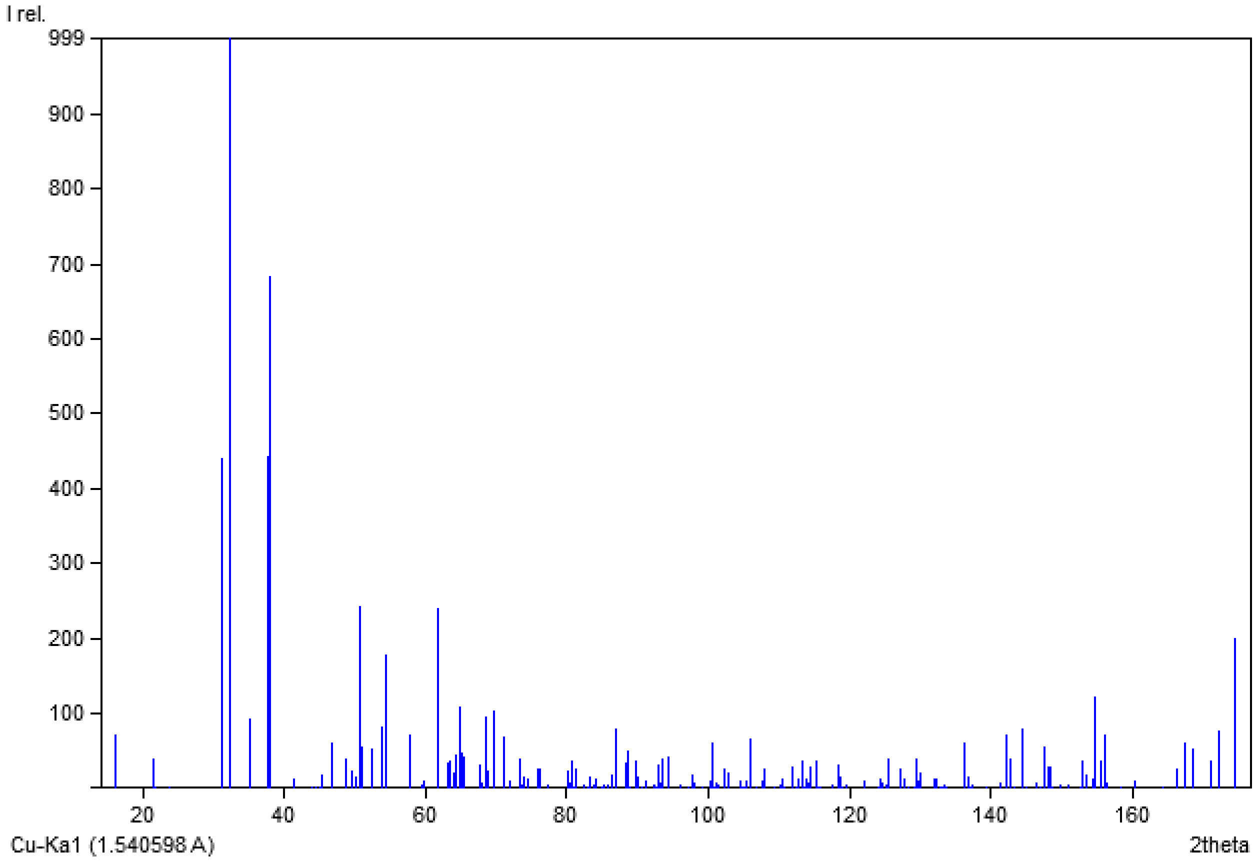

Experimental

Physical Properties

**Calc. density**	8.40500 g/cm³

Remarks

Remarks

**Comments**	- Diffraction pattern calculated by Match!.<br>- I/Ic calculated by Match!.<br>- Space group has been derived from given symmetry operations.
